# Proposing Intelligent Approach to Predicting Air Kerma within Radiation Beams of Medical X-ray Imaging Systems

**DOI:** 10.3390/diagnostics13020190

**Published:** 2023-01-04

**Authors:** Yanjie Lu, Nan Zheng, Mingtao Ye, Yihao Zhu, Guodao Zhang, Ehsan Nazemi, Jie He

**Affiliations:** 1 Department of Digital Media Technology, Hangzhou Dianzi University, Hangzhou 310018, China; 2 College of Pharmacy, Wenzhou Medical University, Wenzhou 325035, China; 3 School of Economics and Management, Zhejiang Ocean University, Zhoushan 316022, China; 4 Department of Physics, University of Antwerp, 2610 Antwerp, Belgium; 5 The First People’s Hospital of Fuyang District, Hangzhou 310000, China

**Keywords:** medical diagnostic radiology, air kerma, RBF neural network, radiology, X-ray beam

## Abstract

The air kerma is a key parameter in medical diagnostic radiology. Radiologists use the air kerma parameter to evaluate organ doses and any associated patient hazards. The air kerma can be simply described as the deposited kinetic energy once a photon passes through the air, and it represents the intensity of the radiation beam. Due to the heel effect in the X-ray sources of medical imaging systems, the air kerma is not uniform within the X-ray beam’s field of view. Additionally, the X-ray tube voltage can also affect this nonuniformity. In this investigation, an intelligent technique based on the radial basis function neural network (RBFNN) is presented to predict the air kerma at every point within the fields of view of the X-ray beams of medical diagnostic imaging systems based on discrete and limited measured data. First, a diagnostic imaging system was modeled with the help of the Monte Carlo N Particle X version (MCNPX) code. It should be noted that a tungsten target and beryllium window with a thickness of 1 mm (no extra filter was applied) were used for modeling the X-ray tube. Second, the air kerma was calculated at various discrete positions within the conical X-ray beam for tube voltages of 40 kV, 60 kV, 80 kV, 100 kV, 120 kV, and 140 kV (this range covers most medical X-ray imaging applications) to provide the adequate dataset for training the network. The X-ray tube voltage and location of each point at which the air kerma was calculated were used as the RBFNN inputs. The calculated air kerma was also assigned as the output. The trained RBFNN model was capable of estimating the air kerma at any random position within the X-ray beam’s field of view for X-ray tube voltages within the range of medical diagnostic radiology (20–140 kV).

## 1. Introduction

The transfer of photon energy to matter occurs in two stages. In the first stage, due to the interaction of the photon with the matter, the energy is transferred to the charged particles of the matter. In the subsequent step, ionized and excited atoms perform the deposition of the kinetic energy of the charged particles. The kerma is equal to the total kinetic energy of the charged particles over the mass of matter [1]. The definition of the kerma is not only limited to the photons but also includes all the uncharged ionizing radiation. The unit of kerma is the gray (Gy), which is equal to the joule/kg, and is the same as the quantity of the absorbed dose. It should be noted that the air kerma is equal to the amount of the kerma of a certain mass of air. Because measuring the amount of air kerma is simpler than measuring the dose quantity, the amount of air kerma is usually used for the calibration of radiation devices [2]. In interventional radiology, the probability of a patient’s skin burning due to intense radiation is high, and so the prediction of the skin dose and the air kerma calculation are of high importance [3]. Researchers’ attention has recently been drawn to the estimation of the air kerma generated by X-ray tubes. In [4], the effect of the anode inclination and X-ray source ripple voltage on the air kerma was studied. In this research, the authors state that by keeping the source voltage constant and increasing the anode angle, the air kerma increases. They also state that the air kerma decreases with the increase in the ripple voltage. In [5], the authors introduce a spectral stripping method using the Monte Carlo simulator for sodium iodide detectors to measure the air kerma. In the absence of the proposed spectrum stripping method, the difference between the received spectrum and reference spectrum was more than 63%, and with the use of the proposed method, this difference was less than 0.2%. The inlet surface air kerma of the chests of pediatric patients was studied by Porto et al. [6]. The key achievement of this investigation was demonstrating the reduction in the air kerma by increasing the voltage and decreasing the exposure. In [7,8,9,10,11,12,13,14], the amount of air kerma was calculated and reported for both the industry and medicine areas. In the abovementioned research, the focus was only on the determination of the air kerma in the center of the X-ray beam. It is important to note that, at a certain distance from the anode, the amount of air kerma varies in different angles within the X-ray beam. This deviation is due to the phenomenon of the anode heel effect.

In the current research, an attempt was made to provide a method for predicting the amount of air kerma despite the phenomenon of the anode heel effect. For this purpose, the Monte Carlo N Particle X version (MCNPX) code was used to simulate and calculate the air kerma at different distances from the source, and at six different X-ray tube voltages. Then, by using the radial basis function (RBF) neural network and training it with limited and discrete data obtained from the MCNPX code, the amount of air kerma was predicted. The trained neural network can calculate the air kerma for a wide range of X-ray source energies at every point within a radiation beam. The significant contributions of the current research are as follows:

1-. The determination of the air kerma by considering the heel effect;
2-. The determination of the air kerma using an artificial neural network, and the training of it with limited data at different angles, distances, and tube voltages;
3-. The calculation of the air kerma at a very high speed in comparison with previous works, and with a very high accuracy, using an artificial neural network;
4-. The determination of the air kerma for the tube voltages used in medical applications.

The current article is structured as follows. First, the medical imaging system simulated by the MCNPX code is thoroughly explained. Then, the training of the RBF neural network using simulation data is discussed. In Section 4, the results and conclusion, respectively, are presented in detail.

## 2. Methodology

### 2.1. Modeling Tube of Medical X-ray Imaging System

**Figure 1:**
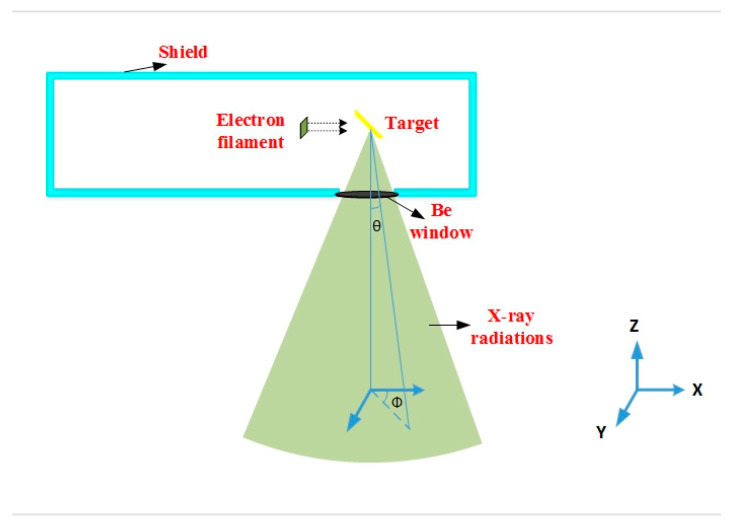
Schematic view of modeled X-ray tube.

To investigate the air kerma within the radiation field of a medical imaging system, only an X-ray source was modeled (see Figure 1). The MCNPX code was implemented to simulate a medical X-ray tube. In many studies [15,16,17,18,19,20], the MCNPX code has been used in the design and simulation of radiation-based systems. To model the electron filament, an electron rectangular surface source with a length and width of 1 mm by 1 mm was considered. The reason for using a surface source instead of a point one was to also model the focal spot. A thin cube made of tungsten with a density of 19,290 kg/m^3^ was placed in front of the electron source inside the tube vacuum chamber as the target. The modeled target had an inclination of 20° in relation to the perpendicular line of the electron-source-target axis. It should be noted that the generated X-ray radiations came out of the tube within a conic with a maximum angle of the X-ray tube’s anode. The electron and target were surrounded by a steel layer to model the vacuum chamber. Just a circular portion on the vacuum chamber was left open against the target. A window made of beryllium with a thickness of 1 mm and density of 1850 kg/m^3^ was positioned at the vacuum chamber’s opening. In the Monte Carlo simulation with a personal computer, including an Intel(R) Core(TM) i7 CPU and 8 GB RAM, the calculation of the air kerma map for a given set of parameters took almost 96 h. 

A point detector tally was employed to measure the air kerma in two steps. Initially, the flux of the photons was measured in each detector. In the second step, the air kerma was calculated using the flux-to-air kerma by converting the factors provided by the International Commission on Radiological Protection ICRP-51 [21] report. The overall statistical uncertainty was retained up to less than 4% in all the simulations. 

Due to the inherent spherical symmetry of the generated X-ray radiations (geometrical wise), a spherical coordinate system was used in this study for placing the point detectors. The point detectors were placed at five various distances from the source: 25 mm, 50 mm, 75 mm, 100 mm, and 125 mm, and at different tangential (0°–20°, with steps of 2°) and polar (Φ = 0°–360°, with steps of 15°) angles. The air kerma was measured in the positioned point detectors for tube voltages of 40 kV, 60 kV, 80 kV, 100 kV, 120 kV, and 140 kV.

### 2.2. Artificial Neural Network

Many researchers have used artificial neural networks for determining various parameters in the field of radiation-based imaging systems and instruments [22,23,24,25,26,27,28,29,30]. The RBF neural network is a popular and fast-learning type of neural network. In this neural network, the radial basis functions are used as the activation functions. Moreover, it is a feed-forward type and has only three layers [31]. The input layer is only responsible for the distribution of the inputs, and it is a linear layer. The second layer provides a nonlinear layer by using the Gaussian function. The last layer provides a linear combination of Gaussian outputs. This neural network is a suitable option for real-time applications due to its fast learning ability. The equation of the radial basis function used in the second layer of the RBF neural network is as follows [31]:

(1) $$\left(r\right)=\exp\left[-\frac{r^{2}}{2\sigma^{2}}\right]$$

where the distance from the center of the cluster is shown by r, and σ is the bell curve’s width. There are computing units called hidden nodes in the second layer. Each hidden node consists of a central vector (c), which is a parametric vector of a length similar to that of the input vector (x). The Euclidean distance between the c and x is calculated according to the following equation:

(2) $$r_{j}=\sqrt{\sum_{i=1}^{n}{\left(x_{i}-w_{ij}\right)}^{2}}$$

The j^th^ neuron’s output in the hidden layer is obtained as follows:

(3) $$\emptyset_{j}=\exp\left[-\frac{\sum_{i=1}^{n}{\left(x_{i}-w_{ij}\right)}^{2}}{2\sigma^{2}}\right]$$

where W represents the weight. The weights can be obtained by utilizing conventional strategies (for example, the K-Mean algorithm [32]) or methods in light of the Kohonen algorithm [33]. Regardless, the training is carried out supervised, the quantity of the anticipated clusters (k) is prechosen, and these algorithms obtain the most appropriate fit for these bunches. In the implementation stages of neural networks, the data are split into two sections: training data and test data. The neural network is implemented by the test data, and the different parameters of the retina are optimized to reduce the error. After completing the network training procedure, the effectiveness of the network should be evaluated against the data it has not seen. A network that successfully completes this stage shows a proper performance under operational conditions. In this research, about 70% of the available data were utilized for the training and optimization of the network parameters, and the rest of the data were assigned as the input to the neural network in the final evaluation. In this research, MATLAB software was used for training and testing the RBF neural network. Although this software has many toolboxes for training different neural networks, no predesigned toolbox was used in this research, and all the steps were meticulously programmed.

## 3. Results and Discussion

**Figure 2:**
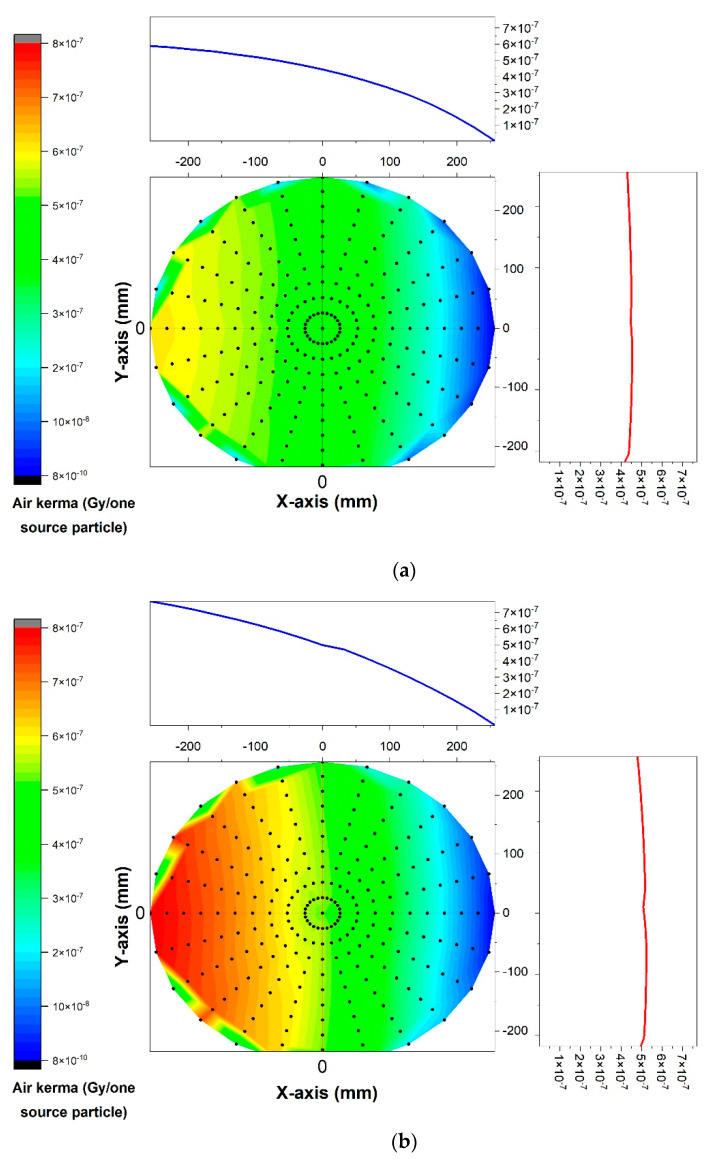
Calculated air kerma within X-ray beam’s field of view of modeled X-ray tube by Monte Carlo simulation for point detectors placed at a certain distance (75 mm) from source of two different tube voltages: (**a**) 60 kV and (**b**) 120 kV.

The calculated air kerma within the X-ray beam’s field of view of the modeled X-ray tube via the Monte Carlo simulation for the point detectors placed at a certain distance (75 mm) from the tube’s anode for two different tube voltages (60 kV and 120 kV) are shown in Figure 2a,b. As can be observed, the calculated air kerma on the right side of the field of view (toward the target) was less than the left side as a result of the heel effect phenomenon, while it remained almost uniform across from the top to the bottom. Moreover, by increasing the voltage, the air kerma increased as well.

**Figure 3:**
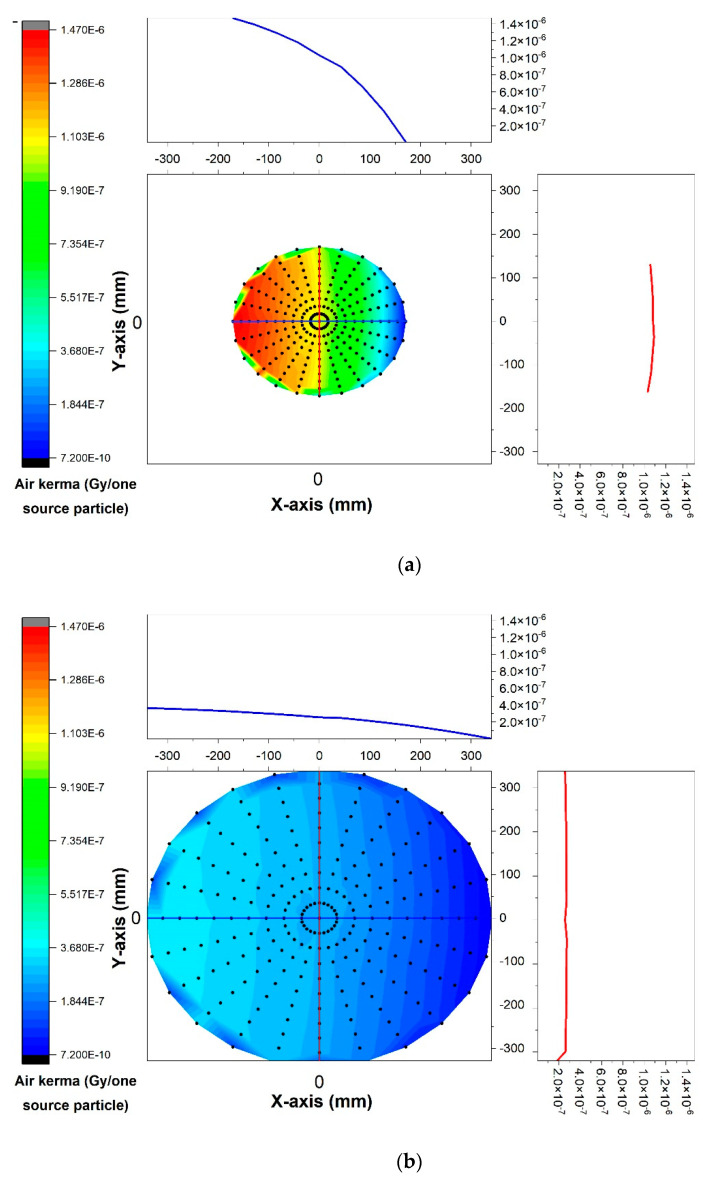
Calculated air kerma when tube voltage was maintained at a value of 80 kV and detectors were positioned at distances of (**a**) 500 mm and (**b**) 1000 mm.

To demonstrate the effect of the distance from the source, the calculated air kerma values when the tube voltage was maintained at a value of 80 kV and the detectors were placed at distances of 500 mm and 1000 mm from the source are shown in Figure 3a,b. As expected, the air kerma was drastically reduced by increasing the distance from the source.

**Figure 4:**
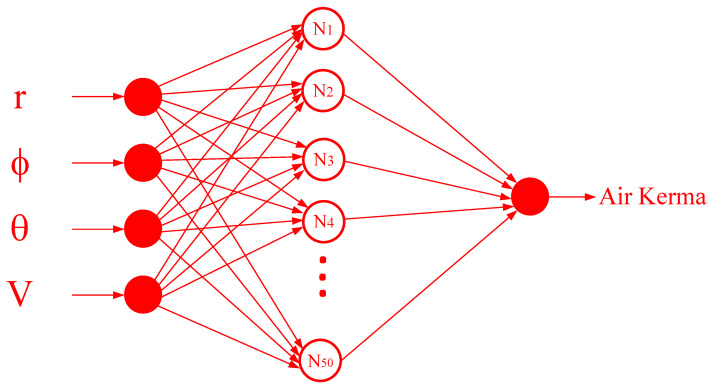
Structure of trained neural network.

**Figure 5:**
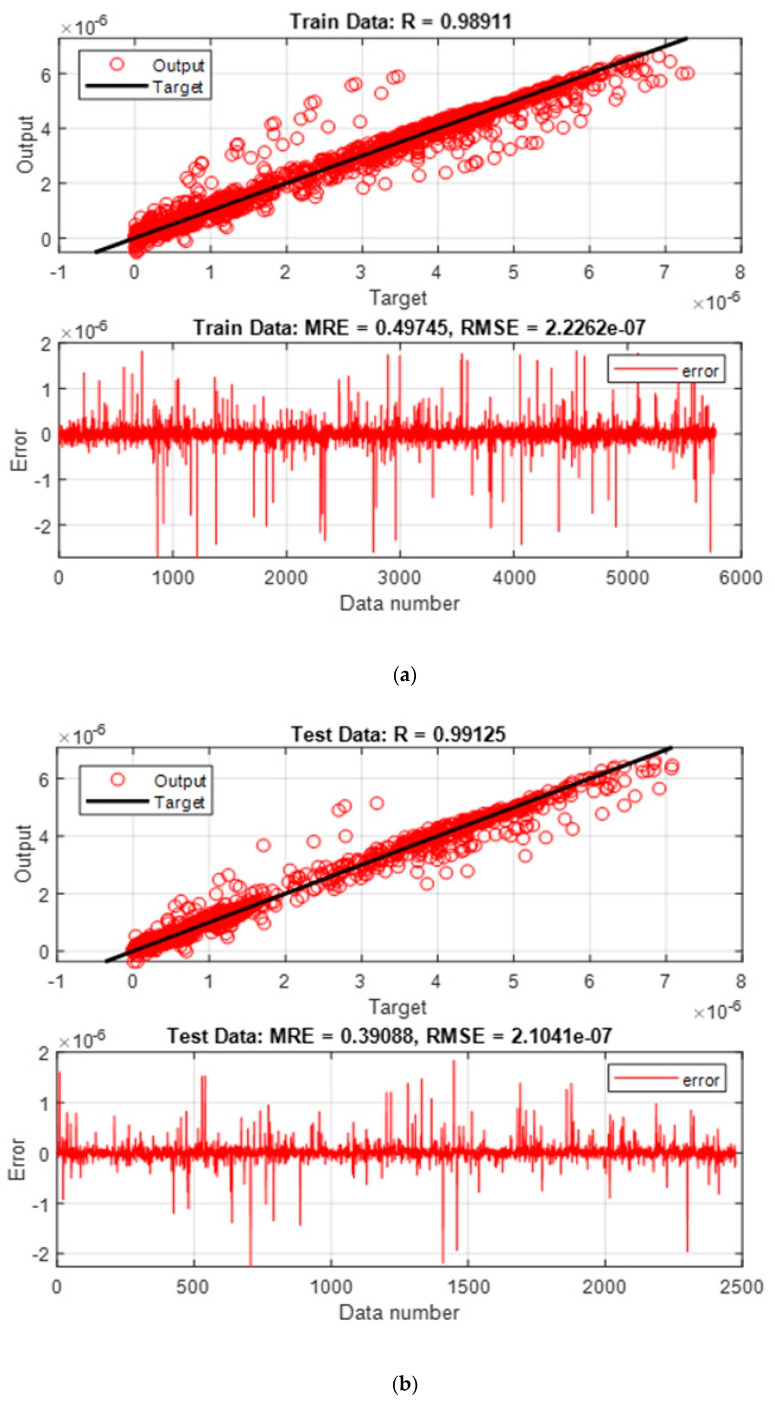
Regression and error diagram showing performance of RBF neural network for (**a**) training and (**b**) test datasets.

Three parameters (ф, θ, and r) that show the location in the radiation field, along with the X-ray tube voltage, were assigned as the inputs of the network, and the output of the network was the amount of air kerma. A total of 5775 samples were randomly selected from the available data to train the neural network. The rest of the data were used for the final analysis of the network after completing the training steps. Because of its advantages, such as fast and supervised learning, a simple structure, and the ability to solve nonlinear problems with high accuracy, an RBF neural network was used in this research. The network included 50 neurons in the hidden layer, which were able to provide the precise relationship between the inputs and output. Figure 4 displays the trained network’s structure. Two error measurement criteria, the root mean square error (RMSE) and mean relative error (MRE), were implemented to calculate the difference between the amount of air kerma obtained from the MCNPX code and the amount of air kerma predicted by the neural network. The equations of these criteria are shown below:

(4) $$MRE\%=100\times \frac{1}{N}\sum_{j=1}^{N}\left|\frac{X_{j}\left(Exp\right)-X_{j}\left(Pred\right)}{X_{j}\left(Pred\right)}\right|$$

(5) $$RMSE={\left[\frac{\sum_{j=1}^{N}{\left(X_{j}\left(Exp\right)-X_{j}\left(Pred\right)\right)}^{2}}{N}\right]}^{0.5}$$

where *N* is the number of data, and X (Exp) and X (Pred) stand for the experimental and predicted values, respectively. To graphically demonstrate the network’s performance, regression and error graphs were drawn for the training and testing data (Figure 5). In the regression diagram, the amount of air kerma acquired by the MCNPX code is displayed as the target output with a black line, and the red circles are the output predicted by the neural network. The matching of these two indicates the high accuracy of the designed network. The error graph shows the difference between the air kerma calculated by the MCNPX code and the air kerma predicted by the neural network.

## 4. Conclusions

In this research, initially, a diagnostic imaging system was modeled with the help of the MCNPX code. Second, the air kerma was calculated at 1375 various discrete positions within the conical X-ray beam for tube voltages in the range of 40–140 kV. In total, the obtained data matrix included four rows, three characteristics related to the location of each point detector and the X-ray voltage, and 8250 columns (various samples) that were used for the neural network implementation. An RBF neural network, which is a supervised and fast learning network, was trained to provide a model for predicting the air kerma based on the location of each point within a beam and the voltage of the X-ray tube. The presented model was able to predict the air kerma with an MRE of no more than 0.50%. It is a very suitable solution for calculating the air kerma due to its high accuracy and high speed. Although the current research tried to predict the air kerma for specific models of X-ray tubes (a fixed target angle of 20°), this methodology can be used to determine the air kerma within the radiation beams of any type of X-ray source. In addition, the presented methodology can be used to determine other radiation parameters, such as the absorbed dose.

## Data Availability

The data presented in this study are available upon request from the corresponding author.

## References

[1] Cember H., Johnson T.E. (2009). Introduction to Health Physics.

[2] IAEA (2000). Calibration of Radiation Protection. Monitoring Instruments.

[3] Miller D.L., Balter S., Schueler B.A., Wagner L.K., Strauss K.J., Vano E. (2010). Clinical radiation management for fluoroscopically guided interventional procedures. Radiology.

[4] Katoh Y., Mita S., Fukushi M., Nyui Y., Abe S., Kimura J. (2011). Calculation of airkerma rate of diagnostic X-ray generators. Radiol. Phys. Technol..

[5] Oliveira L.S.R., Conti C.C., Amorim A.S., Balthar M.C.V. (2013). NaI (Tl) scintillator detectors stripping procedure for air kerma measurements of diagnostic X-ray beams. Nucl. Instrum. Methods Phys. Res..

[6] Porto L., Lunelli N., Paschuk S., Oliveira A., Ferreira J.L., Schelin H., Miguel C., Denyak V., Kmiecik C., Tilly J. (2014). Evaluation of entrance surface air kerma in pediatric chest radiography. Radiat. Phys. Chem..

[7] Alonso T.C., Mourão Filho A.P., Da Silva T.A. (2018). Measurements of air kerma index in computed tomography: A comparison among methodologies. Appl. Radiat. Isot..

[8] Haba T., Koyama S., Aoyama T., Kinomura Y., Ida Y., Kobayashi M., Kameyama H., Tsutsumi Y. (2016). Pin-photodiode array for the measurement of fan-beam energy and air kerma distributions of X-ray CT scanners. Phys. Med..

[9] Kwon D., Little M.P., Miller D.L. (2011). Reference air kerma and kerma-area product as estimators of peak skin dose for fluoroscopically guided interventions. Med. Phys..

[10] Bushberg J.T., Seibert J.A., Leidholdt E.M., Boone J.M. (2012). The Essential Physics of Medical Imaging.

[11] Perera H., Williamson J.F., Li Z., Mishra V., Meigooni A.S. (1994). Dosimetric Characteristics, Air-Kerma Strength Calibration and Verification of Monte Carlo Simulation for a New Ytterbium-169 Brachytherapy Source. Int. J. Radiat. Oncol. Biol. Phys..

[12] Oliveira C., Salgado J., de Carvalho A.F. (2000). Dose rate determinations in the Portuguese Gamma Irradiation Facility: Monte Carlo simulations and measurements. Radiat. Phys. Chem..

[13] Liu Y., Wei B., Zhuoa R., Wena D., Ding D., Xu Y., Mao B. (2016). Determination of the Conventional True Value of Gamma-Ray Air Kerma in a Mini-type Reference Radiation. Appl. Radiat. Isot..

[14] Ounoughi N., Mavon C., Belafrites A., Fromm M. (2015). Spatial distribution of air kerma rate and impact of accelerating voltage on the quality of an ultra-soft X-ray beam generated by a cold cathode tube in air. Radiat. Meas..

[15] Nazemi E., Roshani G.H., Feghhi S.A.H., Setayeshi S., Zadeh E.E., Fatehi A. (2016). Optimization of a method for identifying the flow regime and measuring void fraction in a broad beam gamma-ray attenuation technique. Int. J. Hydrogen Energy.

[16] Roshani G.H., Hanus R., Khazaei A., Zych M., Nazemi E., Mosorov V. (2018). Density and velocity determination for single-phase flow based on radiotracer technique and neural networks. Flow Meas. Instrum..

[17] Roshani G.H., Nazemi E., Roshani M.M. (2017). Intelligent recognition of gas-oil-water three-phase flow regime and determination of volume fraction using radial basis function. Flow Meas. Instrum..

[18] Roshani G.H., Roshani S., Nazemi E., Roshani S. (2018). Online measuring density of oil products in annular regime of gas-liquid two phase flows. Measurement.

[19] Nazemi E., Feghhi S.A.H., Roshani G.H., Peyvandi R.G., Setayeshi S. (2016). Precise void fraction measurement in two-phase flows independent of the flow regime using gamma-ray attenuation. Nucl. Eng. Technol..

[20] Hosseini S., Taylan O., Abusurrah M., Akilan T., Nazemi E., Eftekhari-Zadeh E., Bano F., Roshani G.H. (2021). Application of Wavelet Feature Extraction and Artificial Neural Networks for Improving the Performance of Gas–Liquid Two-Phase Flow Meters Used in Oil and Petrochemical Industries. Polymers.

[21] Nazemi E., Movafeghi A., Rokrok B., Dastjerdi M.C. (2019). A novel method for predicting pixel value distribution non-uniformity due to heel effect of X-ray tube in industrial digital radiography using artificial neural network. J. Nondestruct. Eval..

[22] Sattari M.A., Roshani G.H., Hanus R., Nazemi E. (2021). Applicability of time-domain feature extraction methods and artificial intelligence in two-phase flow meters based on gamma-ray absorption technique. Measurement.

[23] Sattari M.A., Roshani G.H., Hanus R. (2020). Improving the structure of two-phase flow meter using feature extraction and GMDH neural network. Radiat. Phys. Chem..

[24] Mayet A.M., Alizadeh S.M., Nurgalieva K.S., Hanus R., Nazemi E., Narozhnyy I.M. (2022). Extraction of Time-Domain Characteristics and Selection of Effective Features Using Correlation Analysis to Increase the Accuracy of Petroleum Fluid Monitoring Systems. Energies.

[25] Roshani G.H., Nazemi E., Feghhi S.A.H., Setayeshi S. (2015). Flow regime identification and void fraction prediction in two-phase flows based on gamma ray attenuation. Measurement.

[26] Mayet A.M., Salama A.S., Alizadeh S.M., Nesic S., Guerrero J.W.G., Eftekhari-Zadeh E., Nazemi E., Iliyasu A.M. (2022). Applying Data Mining and Artificial Intelligence Techniques for High Precision Measuring of the Two-Phase Flow’s Characteristics Independent of the Pipe’s Scale Layer. Electronics.

[27] Roshani M., Sattari M.A., Ali P.J.M., Roshani G.H., Nazemi B., Corniani E., Nazemi E. (2020). Application of GMDH neural network technique to improve measuring precision of a simplified photon attenuation based two-phase flowmeter. Flow Meas. Instrum..

[28] Roshani G.H., Feghhi S.A.H., Mahmoudi-Aznaveh A., Nazemi E., Adineh-Vand A. (2014). Precise volume fraction prediction in oil–water–gas multiphase flows by means of gamma-ray attenuation and artificial neural networks using one detector. Measurement.

[29] Karami A., Roshani G.H., Khazaei A., Nazemi E., Fallahi M. (2020). Investigation of different sources in order to optimize the nuclear metering system of gas–oil–water annular flows. Neural Comput. Appl..

[30] Alanazi A.K., Alizadeh S.M., Nurgalieva K.S., Nesic S., Grimaldo Guerrero J.W., Abo-Dief H.M., Eftekhari-Zadeh E., Nazemi E., Narozhnyy I.M. (2022). Application of Neural Network and Time-Domain Feature Extraction Techniques for Determining Volumetric Percentages and the Type of Two Phase Flow Regimes Independent of Scale Layer Thickness. Appl. Sci..

[31] Hartman E.J., Keeler J.D., Kowalski J.M. (1990). Layered neural networks with Gaussian hidden units as universal approxima-tors. Neural Comput..

[32] Ralambondrainy H. (1995). A conceptual version of the k-means algorithm. Pattern Recognit. Lett..

[33] Cottrell M., Rousset P. (1997). The Kohonen algorithm: A powerful tool for analysing and representing multidimensional quantitative and qualitative data. International Work-Conference on Artificial Neural Networks.

